# Factors Associated with Worsening Interstitial Fibrosis/Tubular Atrophy in Lupus Nephritis Patients Undergoing Repeat Kidney Biopsy

**DOI:** 10.21203/rs.3.rs-3867933/v1

**Published:** 2024-01-23

**Authors:** Daming Shao, Alejandra Londoño Jimenez, Maria Salgado Guerrero, Shudan Wang, Anna Broder

**Affiliations:** Jacobi Medical Center; McFarland Clinic; University of Alabama at Birmingham; Montefiore Medical Center; Hackensack University Medical Center

**Keywords:** lupus nephritis, repeat kidney biopsy, tubulointerstitial damage

## Abstract

**Background:**

Lupus nephritis (LN) is one of the most severe manifestations of systemic lupus erythematosus (SLE). Interstitial fibrosis/tubular atrophy (IFTA) on kidney biopsies strongly predicts progression to end-stage renal disease. However, factors associated with progression of IFTA are not known. The objective of this study was to evaluate the demographic, clinical, and histopathological factors at the time of index kidney biopsies that are associated with worsening IFTA on repeat biopsies.

**Methods:**

Patients with LN Class I to V or mixed LN on index biopsies who underwent a clinically indicated repeat biopsy between 2004 and 2020 were identified. None-mild IFTA was defined as < 25% acreage of the interstitium affected by fibrosis and atrophy, and moderate-severe IFTA was defined as ≥ 25% of the interstitium affected. Patients with none-mild IFTA on index biopsies who progressed to moderate-severe IFTA on repeat biopsies were defined as progressors. Patients with none-mild IFTA on both biopsies were defined as non-progressors.

**Results:**

Seventy-two patients who underwent clinically indicated repeat kidney biopsies were included, and 35 (49%) were identified as progressors. Compared to non-progressors, progressors had a higher proportion of proliferative LN (20 [57%] vs. 6 [17%], p = 0.002) and crescents (9 [26%] vs. 3 [8%], p = 0.045) on index biopsies. There was no difference regarding the time to repeat biopsy or the baseline characteristics, including eGFR, presence of hypertension and diabetes, urine protein to creatinine ratio, or the initial treatments.

**Conclusions:**

Proliferative LN and the presence of crescents on index biopsies were associated with subsequent IFTA progression on repeat biopsies. This association indicates that glomerular damage is one of the major drivers of tubulointerstitial scarring in SLE. IFTA progression may, in turn, be the driving factor of poor treatment response and progression to chronic kidney disease.

## INTRODUCTION

Lupus nephritis (LN) is one of the most severe manifestations of systemic lupus erythematosus (SLE). Approximately 10 to 30% of patients with LN progress to end-stage kidney disease (ESKD) within ten years despite the prompt use of immunosuppressive therapies [1–4]. Although the current 2003 International Society of Nephrology and Renal Pathology (ISN/RPS) LN classification is primarily based on glomerular histopathology, there is an increased awareness of the prognostic value of tubulointerstitial lesions and an initiative to include interstitial lesions in future LN classification criteria [1–4].

Previous studies have shown that interstitial fibrosis and tubular atrophy (IFTA) is an independent risk factor for worsening kidney function, ESKD progression, and mortality regardless of glomerular damage [5–7]. It has been reported that up to 55% of patients with LN have worsened IFTA on a clinically indicated repeat kidney biopsy [7–10]. However, the histopathological activity in LN patients does not always match the clinical disease activity. Data have shown that many LN patients have histologically active kidney disease with interstitial scarring despite being in clinical remission [11–13] [14], and it is not known whether any clinical or histological characteristics on the initial biopsy are associated with severe IFTA on repeat biopsies.

We aimed to further understand IFTA progression in LN and identify high-risk patients and characteristics that might warrant an early repeat kidney biopsy to monitor IFTA progression and to implement more aggressive therapies to minimize the risk of progression of kidney damage. The objective of this study was to evaluate the demographic, clinical, and histopathological factors of LN patients at the time of index biopsies that are associated with the progression from none-mild IFTA on index kidney biopsies to moderate-severe IFTA on clinically indicated repeat kidney biopsies.

## MATERIALS AND METHODS

### Study population

We identified patients with SLE based on the 1997 American College of Rheumatology (ACR) or the Systemic Lupus Erythematosus International Collaborating Clinics (SLICC) criteria who underwent an index kidney biopsy between 2004 and 2020 at Montefiore Medical Center and Jacobi Medical Center, Bronx, New York. Patients with at least two biopsies showing LN Class I, II, III, IV, V, or mixed LN based on the 2003 International Society of Nephrology (ISN) and the Renal Pathology Society (RPS) classification were included. Patients or the public were not involved in the design, conduct, reporting, or dissemination plans of this research. Indications for a repeat kidney biopsy included worsening proteinuria and serum creatinine levels despite guideline-recommended treatment, an abrupt elevation of serum creatinine level or proteinuria despite initial response to therapy and active lupus flare with suspecting kidney involvement or LN class transformation.

### Demographic and clinical data

Medical charts were reviewed to obtain demographic information, laboratory, and histopathological data at the time of the index biopsy. Data obtained from chart review included age, sex, self-reported ethnicity (analyzed as Hispanic or Latino versus non-Hispanic or Latino), self-reported race (analyzed as Black or African American versus other), body mass index (BMI), history of hypertension and diabetes mellitus at the time of index/first biopsy, the time between lupus diagnosis and index/first biopsy, the interval between index/first biopsy and repeat biopsy, and prior history of antiphospholipid syndrome.

Laboratory and serology data included serum albumin level, estimated glomerular filtration rate (eGFR) by CKD EPI criteria [15], serum complement C3, C4, and double-strand DNA (dsDNA) level at the time of index biopsy. Low serum C3 level was defined as C3 < 80 mg/dL, and low C4 level was defined as C4 < 20 mg/dL. Urine studies collected are the presence of hematuria (defined as urine red blood cell > 5 per high power field), pyuria (defined as urine white blood cell > 5 per high power field), and urine protein to creatinine ratio (UPCR) at the time of index biopsy.

The use of the following medications at the time of the index biopsy was recorded: pulse dose corticosteroid (pulse dose defined as 1 gram daily of intravenous methylprednisolone for three to five days), accumulated steroid dose (defined as total steroid dose in 14 days after the index/first biopsy, dosed as prednisone equivalent), hydroxychloroquine, angiotensin-converting enzyme inhibitor (ACEI) or angiotensin II receptor blocker (ARB), mycophenolate mofetil (MMF), cyclophosphamide, azathioprine, a calcineurin inhibitor (tacrolimus or cyclosporine), and other medications such as belimumab, rituximab, and methotrexate.

### Histopathological data

Histopathological data were obtained from biopsy reports (both the index biopsy and repeat biopsy): ISN/RPS 2003 classification, presence/absence of segmental lesions/crescents/mesangial cellularity/fibrinoid necrosis/hyaline deposition, tubulointerstitial inflammation (TII) severity, and presence/absence of positive C3 and C1q staining on immunofluorescence (IF). LN classes on index/first biopsy were analyzed as proliferative (class III or IV LN), non-proliferative (class I, II, or V), and mixed (class III + V or class IV + V) as treatment and prognosis differ among these groups. IFTA severity was analyzed as none-mild (< 25% of the acreage of interstitium affected based on pathology categorization) vs. moderate-severe (> 25% of the acreage of interstitium affected) [7, 9]. Similarly, the severity of TII was analyzed as none-mild and moderate-severe. Data readily available in all the standard biopsy reports was collected to make this study more generalizable and applicable in clinical practice.

### Statistical analysis

Patients with none-mild IFTA on index biopsy and moderate-severe IFTA on repeat biopsy were defined as progressors, and patients with none-mild IFTA on both biopsies were defined as non-progressors. Bivariate comparisons were performed between progressors and non-progressors. Continuous variables were described as median with interquartile range [IQR]. Categorical variables were expressed as numbers (%). The Mann-Whitney test was used for continuous variables, and Fisher’s exact test and Chi-square test (when appropriate) were used for categorical variables. STATA software (STATA v. 17.0, College Station, TX, StataCorp LLC) was used to conduct all analyses.

## RESULTS

### Overall Patient characteristics

Of the 216 SLE patients with biopsy-proven LN on index biopsy, 81 underwent a clinically indicated repeat kidney biopsy. Nine patients were excluded due to unavailable or incomplete biopsy reports. Seventy-two patients were included in the study (Table 1). Median (interquartile range, IQR) age was 26.9 (18.6–37.4) years old, 58 (81%) were women, 38 (53%) self-identified as Black or African American, and 30 (42%) self-identified as Hispanic or Latino. Fifteen patients (21%) had a history of hypertension, and four patients had a history of diabetes (6%) at the time of index biopsy. The median time from SLE diagnosis to index biopsy was 1.0 (0.0–4.0) years, and the median time interval between the index and repeat biopsy was 2.9 (1.5–5.2) years.

At the time of index biopsy, the median serum creatinine level was 0.9 (0.7–1.2) mg/dL, with median eGFR by CKD EPI of 107.8 (66.1–124.5) ml/min/1.73m^2^. The median albumin level was 3.2 (2.4–3.6) g/dL. Forty-eight patients (74%) had low C3, and 49 patients (75%) had low C4. The median dsDNA level was 136.6 (45.0–222.3) U/mL, and 20 patients (30%) had positive anti-phospholipid antibodies. The median UPCR at the time of index biopsy was 1.9 (0.7–5.2) g/g, but greater than 10% of the data was missing due to remoteness of the data.

Around the time of index biopsy, 57 patients (90%) received corticosteroids, with 20 patients (32%) receiving a pulse dose steroid. Forty-four patients (68%) were started on hydroxychloroquine. Almost all patients were started on immunosuppressives after the index biopsy, except one with class I LN. Fifty-eight patients (82%) were started on MMF, 23 patients (32%) were started on cyclophosphamide, and 10 (14%) were started on a combination regimen with a calcineurin inhibitor (tacrolimus or voclosporin) and mycophenolate or cyclophosphamide as the induction therapy. Sixty-one patients (86%) were on an ACEI or ARB for blood pressure control and proteinuria.

### Histopathological findings

Of the 72 patients included, 28 patients (39%) had non-proliferative LN, 26 patients (37%) had proliferative LN, and 18 patients (25%) had mixed LN on index biopsy (Table 2). At the same time, 26 patients (36%) had globally sclerotic glomeruli, and 12 patients (17%) had crescents. Moderate-severe TII was found in 7 patients (10%), while moderate-severe IFTA was found in 9 patients (13%).

At the time of repeat biopsy, 14 patients (21%) had non-proliferative LN, 28 patients (41%) had proliferative LN, and 27 patients (40%) had mixed LN (3 patients were excluded due to lack of LN classification on repeat biopsy). At the same time, 57 patients (79%) had globally sclerotic glomeruli, and 24 patients (33%) had crescents. Moderate-severe TII was found in 26 patients (36%) and 43 (60%) with moderate-severe IFTA.

While moderate-severe IFTA was present in only 13% of index biopsies, 60% had evidence of moderate-to-severe IFTA on repeat biopsies, with 49% of patients progressing from none-mild to moderate-severe IFTA. Similarly, the proportion of patients with moderate-severe TII increased from 10–36% on repeat biopsies.

### Comparison of progressors and non-progressors

Among the 72 patients who underwent repeat biopsy, 35 (49%) progressed from none-mild IFTA on index biopsy to moderate-severe IFTA on repeat biopsy and were identified as progressors. The remaining 37 patients were identified as non-progressors (%).

The analysis of demographic and clinical factors associated with progression from none-mild to moderate-severe IFTA is summarized in Table 1. There was no difference between the two groups with regards to age, sex, race, BMI, history of hypertension or diabetes, and the interval between index biopsy and repeat biopsy; for laboratory data, no difference was found in baseline eGFR, complement C3 and C4 level; urine studies didn’t show a statistically significant difference in baseline UPCR level between progressors and non-progressors. When comparing medications at the time of index biopsy, no difference was found in the use of ACEI/ARB, hydroxychloroquine, and all other immunosuppressives except for cyclophosphamide, as progressors are more likely to be on cyclophosphamide for induction therapy at the time of index biopsy than non-progressors (15 [44%] vs 8 [22%], p = 0.04).

The analysis of the baseline histopathological data associated with IFTA progression on subsequent biopsies is summarized in Table 2. Compared to non-progressors, progressors included a higher proportion of patients with proliferative LN (20 [57%] vs. 6 [17%], p = 0.002) compared with non-proliferative and mixed LN. The presence of crescents was also found to be more frequent in the progressor group (9 [26%] vs. 3 [8%], p = 0.045). No difference was identified between progressors and non-progressors with regards to the presence of segmental lesions/mesangial cellularity or expansion/fibrinoid necrosis/hyaline deposition, moderate-severe TII, and presence of positive C3 and C1q staining on IF.

## DISCUSSION

This is the first study to identify an association between proliferative LN on index biopsies and IFTA progression on clinically indicated repeat biopsies. Previous studies have shown an association between proliferative LN and moderate-severe IFTA on a single kidney biopsy [4, 6, 16], but no studies to date have demonstrated an association between proliferative LN on index biopsies and IFTA on clinically indicated repeat biopsies. To our knowledge, only two studies by Pagni et al. and Kwon et al. investigated possible factors related to IFTA on repeat biopsy. Pagni et al. found a correlation between class IV LN and severity of interstitial fibrosis on the same repeat biopsies, but the study didn’t evaluate the association between proliferative LN on index biopsies and the progression of IFTA [8]. Kwon et al. found that a high renal component of systemic lupus erythematosus disease activity index (SLEDAI) was associated with IFTA progression, but the study didn’t investigate the association between classification of LN and IFTA progression [9]. The association between proliferative LN on index biopsies and IFTA progression further suggests that patients with LN who develop severe IFTA are more likely to have more severe glomerular disease at the initial presentation.

Prior studies have proposed several possible mechanisms for glomerular-interstitial disease interaction. First, the deposition of immune complexes can occasionally fill the capillary loops and lead to subsequent complement activation, damaging the glomerular microvasculature and causing interstitial ischemia. Second, the increased protein filtration and proteinuria from damaged basal membrane results in inflammation and cytotoxic injury to the tubules and interstitium. Third, some autoimmune antibodies, such as anti-cytoplasmic antibodies that target immunogenic antigen vimentin expressed in LN with tubulointerstitial lesions, might also play a role [17, 18]. Persistent inflammatory damage leads to cytokine release, macrophage activation, increased fibroblast function, and, eventually, IFTA.

While questions remain regarding the complex relationship between glomerular and tubulointerstitial lesions in LN patients, our results are consistent with prior studies showing high rates of worsening tubulointerstitial lesions on clinically indicated repeat biopsies (49% in this study versus previously reported 35–55% rate of progression) [7–10]. Similarly, we did not identify correlations between demographic or laboratory parameters and IFTA progression, and commonly tested biomarkers have been shown to be insufficient to assess LN activity. Apart from clinically indicated repeat biopsies when patients have active or recurrent LN, several prior studies investigating repeat kidney biopsies post-treatment or protocol repeat biopsies have demonstrated a discordance between clinical and histological activity during long-term follow-up, with persistent LN activity including interstitial inflammation and scarring among patients who have achieved a complete renal response to treatment [12–14, 19]. Taken together, these findings underscore the need to identify non-invasive biomarkers associated with IFTA progression.

This study also has several treatment implications. The association between proliferative LN and IFTA progression, together with underlying glomerular-interstitial interaction, provide a strong rationale to consider the use of combination regimens with recently approved agents, voclosporin and belimumab, as the induction treatments in patients with proliferative LN to minimize progression [20, 21]. Currently, a combination regimen with belimumab or voclosporin is recommended for LN patients with inadequate responses after several months of standard induction therapy [22, 23]. The potential use of anti-fibrotic medications targeting pro-fibrotic cytokines and subsequent fibrosis pathways in patients with LN should also be investigated [24].

This study has several limitations due to its retrospective nature and a relatively small sample size. The records of patients enrolled in this study were obtained over a period of more than fifteen years, and clinical data recording was not consistent over time. As a result, some important data, such as indications for each repeat biopsy, SLEDAI, and the NIH activity and chronicity scores, were not consistently available and were not evaluated. For the same reasons, a central review of kidney biopsies with a more detailed evaluation of the IFTA and TII was not performed. The study sample included patients with relatively preserved kidney function at the time of index biopsy. As a result, detecting the significance of several variables might be underpowered due to a relatively small number of progressors in this cohort. The effects of different induction therapies and medication adherence on IFTA progression could also not be assessed due to the retrospective design.

The major strength of this study is the real-world experience of LN patients from two large, urban tertiary care centers with homogeneity of treatment at the time of index biopsy. Another strength is the large proportion of racially and ethnically diverse patients, with the majority of patients self-identifying as Hispanic or Latino and Black or African American.

In conclusion, this study revealed that proliferative LN was associated with IFTA progression on repeat kidney biopsy and identified LN patients who are at higher risk of tubulointerstitial scarring. Our study also provides insight into the complex relationship between glomerular and tubulointerstitial disease progression in patients with LN and lays the foundation for future prospective studies investigating novel markers of interstitial damage and worse kidney outcomes.

## Figures and Tables

**Table 1:** Factors associated with progression from none-mild to moderate-severe IFTA

Factors	Overall N = 72	Progressors N = 35	Non-progressors N = 37	*P*-value
**Demographics and comorbidity data at index/first biopsy**				
Age, median (IQR), years	26.9 (18.6–37.4)	28.4 (20.8–37.6)	24.0 (17.2–32.1)	0.30
Female, n(%)	58 (80.6)	28 (80.0)	30 (81.1)	0.91
Hispanic ethnicity, n(%)	30 (41.7)	18 (54.6)	12 (32.4)	0.06
African American Race, n(%)	38 (52.8)	13 (37.1)	25 (67.6)	0.07
BMI, median (IQR), kg/m^2^	24.9 (21.5–29.8)	25.9 (23.0–31.6)	22.7 (21.2–28.9)	0.18
History of Hypertension, n(%)	15 (20.8)	9 (25.7)	6 (16.2)	0.20
History of Diabetes, n(%)	4 (5.6)	3 (8.6)	1 (2.7)	0.06
Years between SLE diagnosis and index/first biopsy,	1.0 (0.0–4.0)	2.5 (0.0–4.0)	1.0 (0.0–4.0)	0.24
median (IQR), years				
Years between index/first biopsy and repeat biopsy,	2.9 (1.5–5.2)	2.5 (1.4–5.2)	3.2 (2.0–4.6)	0.62
median (IQR), years				
History of Antiphospholipid syndrome, n(%)	6 (9.1)	2 (6.7)	4 (11.1)	0.53
**Laboratory data at index/first biopsy**				
Serum Albumin level, median (IQR), g/dL	3.2 (2.4–3.6)	3.3 (2.4–3.6)	3.2 (2.6–3.6)	0.92
eGFR by CKD EPI, median (IQR), ml/min/1.73m^2^	107.8 (66.1–124.5)	88.3 (60.9–120.3)	115.9 (75.5–128.5)	0.10
Complement C3 level, median (IQR), mg/dL	63.0 (48.0–80.0)	62.0 (51.0–79.0)	65.0 (47.0–90.0)	0.85
Complement C4 level, median (IQR), mg/dL	11.0 (7.0–18.0)	9.5 (7.0–14.0)	9.5 (7.0–14.0)	0.36
dsDNA at index/first biopsy, median (IQR), U/mL		164.7 (76.5–222.3)	98.8 (35.3–227.7)	0.30
Positive anti-phospholipid antibody, n(%)	20 (30.3)	10 (33.3)	10 (27.8)	0.63
**Urine studies at index/first biopsy**				
Hematuria (RBC > = 5/HPF), n(%)	49 (72.1)	22 (71.0)	27 (73.0)	0.85
Pyuria (WBC > = 5/HPF), n(%)	39 (58.2)	19 (63.3)	20 (54.1)	0.44
UPCR, median (IQR), g/day*	1.9 (0.7–5.2)	2.1 (0.9–5.2)	1.8 (0.7–5.2)	0.67
**Medication use at or after index/first biopsy, n(%)**				
Pulse dose steroid, n(%)*	20 (31.7)	12 (34.3)	8 (21.6)	0.16
^+^Average prednisone dose, median (IQR), mg*	560.0 (280.0–840.0)	700.0 (280.0–840.0)	560.0 (280.0–840.0)	0.68
HCQ, n(%)	44 (67.7)	20 (66.7)	24 (68.6)	0.87
ACEi/ARB, n(%)	61 (85.9)	28 (82.4)	33 (89.2)	0.41
Mycophenolate, n(%)	58 (81.7)	30 (88.2)	28 (75.7)	0.17
Azathioprine, n(%)	21 (29.6)	7 (20.6)	14 (37.8)	0.11
Calcineurin inhibitor, n(%)	10 (14.8)	5 (14.7)	5 (13.5)	0.88
Cyclophosphamide, n(%)	23 (32.4)	15 (44.1)	8 (21.6)	0.04
Rituximab, n(%)	4 (5.6)	2 (5.7)	2 (5.4)	0.95

* More than 10% of the data are missing due to the longevity of the records.

+ Average total prednisone equivalent steroid dose 14 days after index/first biopsy.

Abbreviations: BMI, Body mass index; CKD EPI, Chronic Kidney Disease Epidemiology Collaboration equation; UPCR, urine protein to creatinine ratio; HCQ, Hydroxychloroquine; ACEi, Angiotensinconverting enzyme inhibitor; ARB, Angiotensin II receptor blockers.

**Table 2:** Histopathological findings on initial/first biopsy with IFTA progression

Factors		Overall N = 72	Progressors N = 35	Non-progressors N = 37	*P*-value
**Histopathological findings on initial/first biopsy**					
	Proliferative Lupus Nephritis, n(%)	26 (36.1)	20 (57.1)	6 (16.7)	0.002
	ISN/RPS Classifications of Lupus Nephritis				
Non Proliferative Type	Class I	2 (2.8)	0 (0.0)	2 (5.4)	
	Class II	8 (11.1)	2 (5.7)	6 (16.2)	
	Class V	17 (23.6)	7 (20.0)	11 (29.7)	
Proliferative Type	Class III	9 (12.5)	7 (20.0)	2 (5.4)	
	Class IV	17 (23.6)	13 (37.1)	4 (10.8)	
Mixed Type	Mixed (Class III + V or IV + V)	18 (25.0)	6 (17.1)	12 (29.7)	
Presence of globally sclerotic glomeruli, n(%)		26 (36.1)	12 (34.3)	14 (37.8)	0.75
Presence of segmental sclerosis, n(%)		34 (47.2)	17 (48.6)	17 (45.9)	0.82
Presence of crescents, n(%)		12 (16.7)	9 (25.7)	3 (8.1)	< 0.05
Presence of mesangial cellularity, n(%)		49 (68.1)	26 (74.3)	23 (62.2)	0.27
Presence of fibrinoid necrosis, n(%)		3 (4.2)	1 (2.9)	2 (5.4)	0.59
Presence of hyaline deposition, n(%)		10 (13.9)	6 (17.1)	4 (10.8)	0.42
Presence of moderate-severe TII, n(%)		7 (9.7)	4 (11.4)	3 (8.1)	0.64
	Positive C3 staining on IF, n(%)	49 (73.1)	25 (80.6)	24 (66.7)	0.20
	Positive C1q staining on IF, n(%)	18 (26.9)	8 (34.8)	10 (34.5)	0.98

Abbreviations: ISN/RPS, International Society of Nephrology/Renal Pathology Society; TII, tubulointerstitial inflammation; IFTA, interstitial fibrosis/tubular atrophy; IF, immunofluorescence.

## Data Availability

The data underlying this article are available in the article.

## HIGHLIGHTS

- Interstitial fibrosis/tubular atrophy (IFTA) is an independent risk factor for worsening kidney outcomes, but the associated factors of worsening IFTA have remained unclear.
- Proliferative LN and the presence of crescents on the initial biopsy were associated with worsening IFTA on repeat kidney biopsies.
- Glomerular damage is one of the major drivers of tubulointerstitial scarring in SLE.
